# Immediate effects of the incentive spirometer in women with healthy voice

**DOI:** 10.1590/2317-1782/20232022291en

**Published:** 2023-11-10

**Authors:** Bárbara Pereira Lopes, Gustavo Polacow Korn, Flávio Barbosa Nunes, Ana Cristina Côrtes Gama

**Affiliations:** 1 Programa de Pós-graduação em Ciências Fonoaudiológicas, Universidade Federal de Minas Gerais - UFMG - Belo Horizonte (MG), Brasil.; 2 Departamento de Otorrinolaringologia, Faculdade de Medicina, Universidade Federal de São Paulo - UNIFESP - São Paulo (SP) Brasil.; 3 Departamento de Otorrinolaringologia, Faculdade de Medicina, Universidade Federal de Minas Gerais - UFMG - Belo Horizonte (MG), Brasil.; 4 Departamento de Fonoaudiologia, Faculdade de Medicina, Universidade Federal de Minas Gerais - UFMG - Belo Horizonte (MG) Brasil.

**Keywords:** Voice, Voice Training, Respiration, Breathing Exercises, Speech, Language and Hearing Sciences

## Abstract

**Purpose:**

To evaluate the immediate effect of the incentive spirometer on acoustic measures, aerodynamic measures and on the auditory-perceptual assessment of vocal quality in vocally healthy women.

**Methods:**

This is an experimental intra-subject comparison study with the participation of 22 women without vocal complaints. Acoustic measures, aerodynamic measures and auditory-perceptual assessment of vocal quality were obtained before and immediately after using the incentive spirometer by the participants. The device was used in the orthostatic position and the participants performed three sets of ten repetitions with a one-minute interval between sets.

**Results:**

After using the incentive spirometer, there was a significant reduction in jitter, shimmer and PPQ (period perturbation quotient) measurements and an increase in maximum expiratory volume, while the other acoustic and aerodynamic measurements were not significantly impacted. In addition, there was improvement in vocal quality in eight (36.4%) participants and 11 (50.0%) participants showed no changes in the auditory perceptual assessment of voice quality after using the incentive spirometer.

**Conclusion:**

The use of the incentive spirometer is safe and, in its immediate effect, positively impacts the acoustic measures of short-term aperiodicity of frequency and intensity and increases the maximum expiratory volume in women with healthy voices.

## INTRODUCTION

Voice production can be defined as a complex mechanism that involves the sound source, composed of the vocal cords and vocal tract. The vocal tract works as a noise modulation filter, thus being responsible for the resonant and articulatory aspects of voice emission^(1)^. In addition, the sound production in the sound source requires the interaction between two forces: the aerodynamic force, which is the airflow exhaled from the lungs and flowing along the trachea towards the upper airways, and the myoelastic force, which is the medialization movement of the vocal cords to promote resistance to the exhaled air^(1)^.

Such resistance to airflow causes an increase in pressure below the vocal cords. When high, this pressure can break the blockage promoted by the vocal cords in the median position and allow the passage of air between them^(2)^. Soon after this air escape, the infraglottic pressure decreases again, favoring the return of the glottis to the closed state and promoting a vibratory movement of the mucosa covering the vocal cords. This phenomenon is called the Bernoulli Effect, which is the production of matter displacement due to the pressure difference along a segment^(1)^. Thus, aerodynamic energy is converted into acoustic energy, resulting in sound waves that are modulated along the entire vocal tract^(1)^. A glottal cycle takes place each time this event occurs, and the occurrence of several such cycles makes up vocal production^(2)^.

The changes in aerodynamic and myoelastic forces, either isolated or simultaneous, might cause an imbalance of the phonatory system and compromise a healthy voice emission due to the break in homeostasis^(3)^. Therefore, both forces involved in speech must be considered when promoting, preventing, and recovering voice health.

The speech therapist who acts in the voice therapy is responsible for providing treatment of vocal disorders already in place, or even improving adjustments and promoting better conditioning of the muscles involved in voice production^(4)^. For such a purpose, it is fundamental for a successful treatment to use an approach that encompasses not only the laryngeal muscles but also considers the breathing involved in the phonatory process^(5)^.

Over the years, researchers have developed instruments that can assess voice production both objectively and subjectively. The extraction of acoustic and aerodynamic measures through specific software is an example of objective assessments, while the auditory perception of speech and the vocal self-assessment protocols represent subjective evaluations^(6)^. Such assessments supported the establishment of guidelines for speech therapists regarding their applications, thus favoring the understanding of the phonatory system and the identification of unbalance in the interaction between myoelastic and aerodynamic forces in voice production^(6)^.

The voice assessment protocol suggested by the literature^(6,7)^ includes the auditory perception of speech, vocal self-assessment, and instrumental voice assessment; the latter involves laryngeal, breathing aerodynamic, and acoustic voice assessments^(6)^.

Some objective respiratory measures, such as those linked to pressure, volume, and airflow, can be performed using speech therapy assessment since voice production is a physical phenomenon involving such properties and their derivatives^(6,8)^. These measures are performed on specific software and extracted automatically through pre-established tasks by the program, like in the extraction of acoustic measures. Not only can they be useful for checking the integrity of the phonatory system but can also suggest pulmonary pathologies such as asthma^(9)^, in addition to favoring the monitoring of the speech therapy evolution throughout the treatments^(5)^.

Comprising breathing assessment into the voice therapy might highly favor more assertive therapeutic planning since some cases of dysphonia require direct intervention in the aerodynamic process^(5)^. Such a direct approach through breathing training might foster a better prognosis since it benefits the rebalancing of the forces involved in the voice production process^(10)^.

In the speech therapy practice, one of the ways to train the respiratory muscles is with the use of respiratory stimulants, devices that involve the functions of inhalation and/or exhalation depending on the therapeutic objective^(5)^. The literature^(11)^ reports that the use of respiratory stimulants, more specifically the incentive spirometer, can increase the measures of maximum inspiratory and expiratory pressures, which might positively influence even the biomechanics of swallowing.

Even though respiratory stimulants have been largely used in voice therapy, only a few studies have addressed their impact^(12)^, which is the objective of our study. Understanding scientific research not only as an evidence-driven approach but also as an enabler for clinical practice so that the actual related benefits are confirmed is fundamental. Therefore, this study is defined as translational research^(13)^.

The literature^(13)^ classifies experimental research studies in the area of Rehabilitation Science into five phases: 1) Phase 0 encompasses observational studies aimed at defining the prevalence and variables associated with a given clinical condition; 2) Phase 1 refers to experimental studies based on single-subject design without the clinical condition to assess the safety and dose effect of a given clinical intervention; 3) Phase 2 corresponds to experimental studies based on single-subject design with the clinical condition to analyze the effect of the intervention on the subject’s clinical condition; 4) Phase 3 cover experimental studies using Randomized Clinical Trials (RCT) to evaluate the efficacy of a given intervention; and 5) Phase 4 comprises studies on heterogenous populations regarding the effectiveness of a given intervention and its application in public policies^(13)^.

Considering the importance of Phase 1 studies for developing further studies with high evidence levels, our study has the following guiding question: What are the effects and safety of using an isolated incentive spirometer on voice quality and aerodynamic measures of vocally healthy individuals?

Therefore, this study aimed to evaluate the immediate effect of an incentive spirometer on the aerodynamic and vocal measures of vocally healthy women. Our research hypothesis was that the incentive spirometer as a direct respiratory approach is safe and can be used in speech therapy clinical practice. The device has positive effects on voice quality and aerodynamic measures, possibly favoring a better conversion of aerodynamic energy into acoustic energy, hence improving voice performance.

This study is justified by introducing scientific knowledge on the safety and immediate effects of using incentive spirometers on voice therapy, thus supporting further research on their therapeutic efficacy using higher-evidence-level designs, such as Randomized Clinical Trials.

## METHODS

This is an experimental study with intra-subject comparison (Phase 1)^(13)^, approved by the Ethics Research Committee of the Federal University of Minas Gerais – (Universidade Federal de Minas Gerais (UFMG)) (4,331,770). The single-subject design without the clinical condition (Phase 1) was defined based on the need to investigate the safety and effect of Respiron® on the voice quality of vocally healthy women.

Twenty-two women, aged between 18 and 43 years old – mean age of 26 years old (standard deviation 7.28), participated in this study by signing the Informed Consent Form (ICF) (in Portuguese Termo de Consentimento Livre e Esclarecido (TCLE)). All data were collected at the Functional Health Observatory in Speech and Hearing Therapy of UFMG (OSF/UFMG) in an acoustically treated room.

Women with no self-reported vocal complaints and neutral vocal quality (G0) were included in the study. The presence or absence of vocal complaints was assessed on the day of collection by analyzing the self-perception of vocal quality (report having/not having a good or very good voice), which was positive, and by analyzing the presence/absence of vocal symptoms (fatigue and/or discomfort), which was absent. Vocal quality was analyzed by the auditory perception of speech of the general dysphonia grade parameter (G). The auditory perception of speech was performed by consensus between two speech therapists with more than ten years of experience in voice therapy.

The following exclusion criteria were considered: being a singer, being a smoker, having felt discomfort while performing the exercises, and having obtained a score higher than two points on the Dyspnea Scale: Medical Research Council Modified, adapted to Brazilian Portuguese^(14)^.

The Dyspnea Scale is an easy-to-use and understandable instrument that gradually assesses the intensity of dyspnea complaints that impact the performance of activities of daily living^(14)^. In the absence of a dyspnea complaint, the participants were instructed to answer “not applicable”, and in the presence of a complaint, they should indicate, among a total of five items, how it impacts their activities of daily living based on the descriptions of examples offered by the scale^(14)^.

All participants had their emissions recorded in an acoustically treated room. Emissions were processed directly on a Dell® Optiplex GX260 computer equipped with a Direct Sound® professional sound card. A unidirectional condenser microphone (Shure®) was used, positioned five centimeters from the lip commissure, with a 45° directional pickup angle. The signals were recorded at a sampling rate of 44100 Hz, 16 bits of resolution, mono channel, and WAV format (Waveform Audio File Format). The participant was asked to utter the sustained vowel /a/ in an orthostatic position habitually and fully, in addition to counting from one to 20.

### Assessments

**Figure 1:**
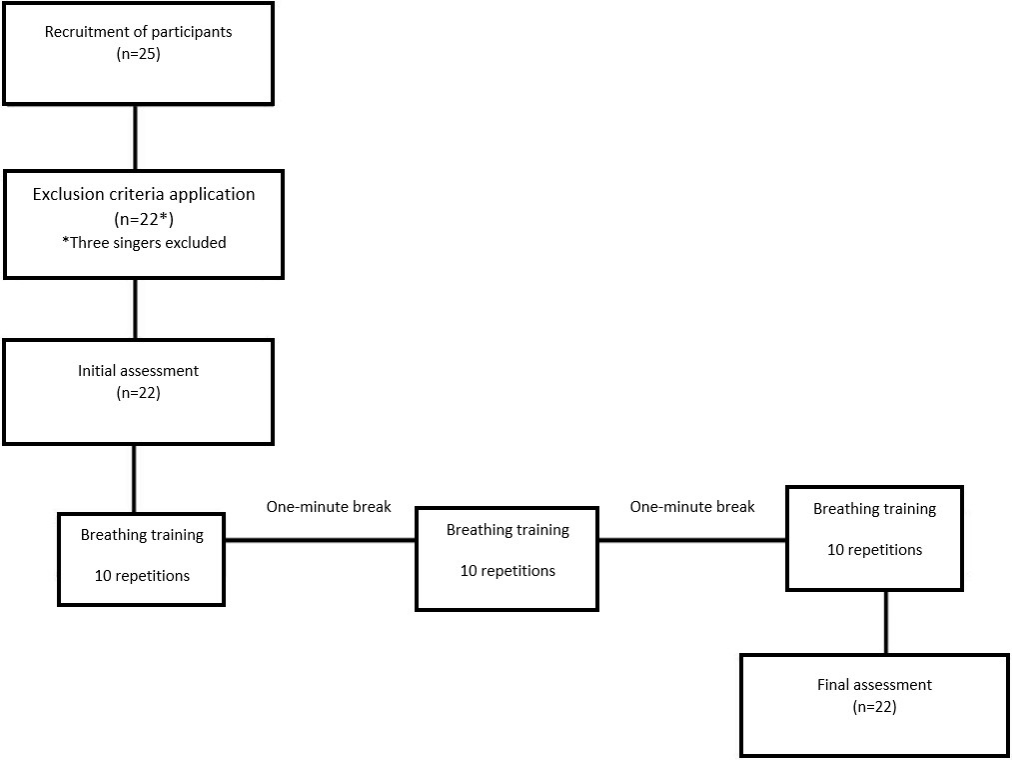
Flowchart of the study steps

Each of the participants underwent two assessments. The first assessment at the time point (M1) was considered the beginning of the period without intervention, the baseline measurement. The second assessment at the time point (M2) was performed after the participants used the Respiron® incentive spirometer (NCS S.A., Barueri, SP, Brazil) to analyze the immediate effect of the technique (Figure 1).

#### Acoustic voice assessment

The acoustic assessment through the emission of vowel /a/ considered the following measures extracted automatically by the Multi-Dimensional Voice Program (MDVP), Kay Pentax®: fundamental frequency, in Hertz; jitter *(%)*, period perturbation quotient (PPQ) (*%*), shimmer *(%)*, and amplitude perturbation quotient *(*APQ) (*%*). These are short-term perturbation measures of the sound signal, the first two refer to frequency perturbations and the other two correspond to intensity perturbations. The noise harmonic ratio (NHR) (dB) contrasts with the harmonic component and the noise component of the produced sound wave. The following cepstral measures were also taken: Cepstral Peak Prominence (CPP) (dB) and Cepstral Peak Prominence-Smoothed (CPPS) (dB) for the emissions of the sustained vowel /a/ and the speech sample obtained from the counting from one to 20 by the Praat software (Paul Boersma and David Weenink, University of Amsterdam, Netherlands).

#### Auditory perception of speech

For the auditory perception of speech, the voices for the M1 and M2 moments, both referring to the sustained vowel and the speech task by counting from one to 20, were divided into pairs randomly for the speech therapist examiners to perform a blind evaluation. For such a purpose, three examiners with experience of over five years in voice therapy were instructed to classify the voice pairs by indicating whether there has been improvement, worsening, or no change in voice quality between the second and first voices. The procedure required the use of supra-aural earphones. Since the voices had been classified as neutral, that is, with a deviation in the general degree of voice quality regarded as zero, the examiners did not need to specify the auditory perception parameter that most supported their decisions.

#### Aerodynamic voice assessment

The aerodynamic measures were assessed using the CSL software by Kay Pentax^TM^, model 6103, Lincoln Park NJ USA, Phonatory Aerodynamic System (PAS) module. The following three tasks were covered: maximum forced exhalation, emission of the vowel /a/ for as long as possible, and repetition of the syllable /pa/ for a minimum of seven consecutive times. The following measures were extracted: maximum expiratory volume (liters, l); maximum speech time (seconds, s); peak air pressure and mean peak air pressure (centimeters of water, cm H_2_O); voice airflow and peak expiratory flow (liters per second, l/s); aerodynamic resistance [centimeters of water divided by the fraction of liters per second, cm H_2_O/ (l/s)]; acoustic impedance (ohms, Ω); aerodynamic force (watts, W), and aerodynamic efficiency (parts per million, p.p.m.)

**Figure 2:**
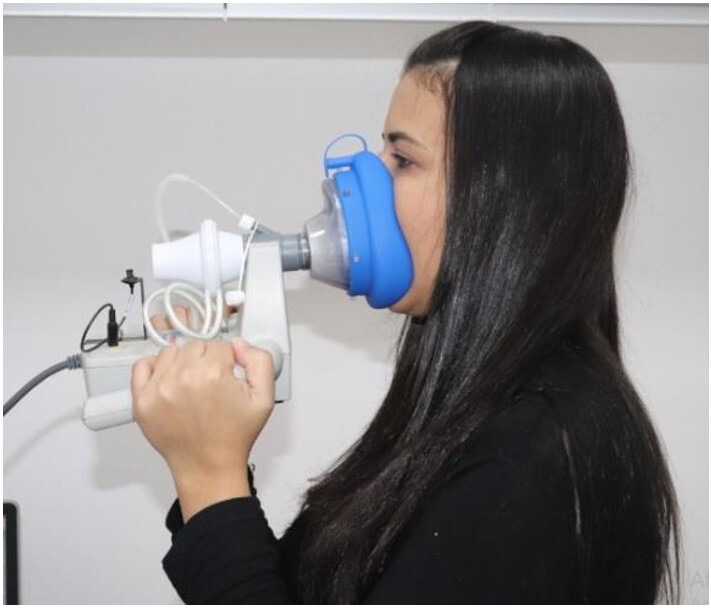
Use of a silicone face mask coupled with a pressure transducer to capture aerodynamic measures.

Aerodynamic measures were captured using a silicone face mask with a small polyethylene catheter positioned under the participant's tongue and coupled to a pressure transducer (Figure 2).

### Use of the incentive spirometer by Respiron®, Classic model

**Figure 3:**
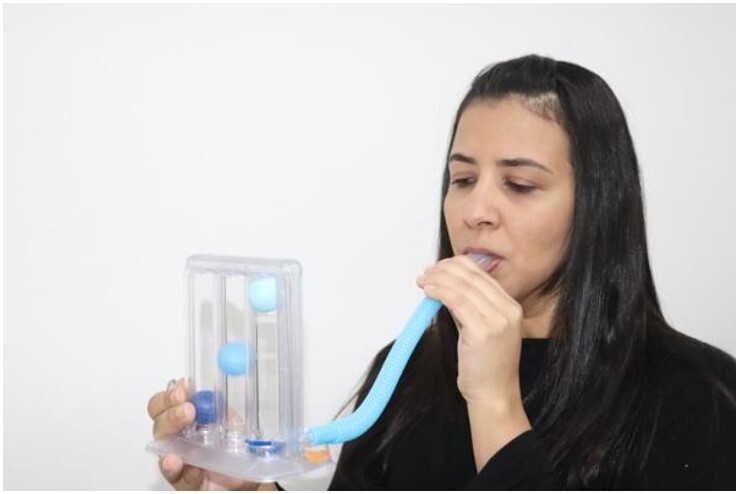
Use of the incentive spirometer by Respiron®, Classic model

The participants were instructed to use the incentive spirometer by Respiron®, Classic model (NCS S.A., Barueri, SP, Brazil), in the orthostatic position (Figure 3). The device contains three spheres with different intensities and can be adjusted to four increasing levels of air resistance. In this case, the device was regulated at the highest level upon self-reported comfort by the participant.

The following instructions were given concerning the use of the device: exhale as much air as possible, then inhale as much as possible orally, followed by inhaling the air orally through the nozzle attached to the device, trying to elevate the three spheres and keep them high for approximately three seconds.

Three series of ten repetitions were performed with a one-minute interval between them. After completing the training, the participants were reassessed through the same procedures used in the initial assessment. The dose of Respiron® (NCS S.A., Barueri, SP, Brazil), which is the number of repetitions, was established based on the number suggested by the manufacturer in the user manual^(15)^. This dose is also reported in the literature^(11)^.

### Data analysis

The data statistical analysis was performed on the MINITAB statistical software, version 17. Firstly, a descriptive data analysis using measures of central trend and dispersion was conducted. Subsequently, the Anderson-Darling test to assess the sample normality was used. The groups were compared through the paired t-test or Wilcoxon non-parametric test. The level of confidence was 95%. The agreement among the examiners regarding the auditory perception of speech was analyzed through Gwet’s AC1 coefficient on the R software, version 3.3.1. The agreement degree was analyzed as follows: values below zero – no agreement; 0 to 0.20 – low agreement; 0.21 to 0.40 – weak agreement; 0.41 to 0.60 – moderate agreement; 0.61 to 0.80 – good agreement, and 0.81 to 1.00 – almost perfect agreement^(16)^. Such an analysis considered the mode value of the answers given by the speech therapist examiners. The three examiners disagreed in only two voice pairs. In these cases, a fourth examiner, a voice specialist speech therapist with over 20 years of experience, evaluated the two voice pairs to establish the most agreed answer.

## RESULTS

To perform the guided task, the incentive spirometer was adjusted according to the highest level at which the participant showed self-reported comfort. To this end, a test was repeated at the zero level of the device, which also occurred in the ascending levels until the maximum level of self-reported comfort. However, even in the presence of self-reported comfort, when there was an exaggerated contraction in the cervical musculature, the researchers chose to return to the previously tested level to avoid vocal damage due to excessive musculoskeletal stress. Thus, respecting the level of comfort in performing the task, 12 participants (54.6%) used the device set at level zero, five participants (22.7%) at level one, and five (22.7%) at level two.

The analyses of the acoustic measures before and after the use of the incentive spirometer showed an improvement in the parameters of short-term frequency (jitter and PPQ) and short-term intensity aperiodicity (shimmer) (Table 1).

**Table 1:** Acoustic measures in the moments before (M1) and immediately after (M2) use of the incentive spirometer (N = 22)

		Mean	SD	Median	*p*-value
**F0 (Hz)**	M1	218.49	27.50	218.95	0.171*
	M2	224.75	27.99	222.83	
**Jitter (%)**	M1	1.684	0.931	1.461	˂0.001**
	M2	1.147	0.759	1.000	
**Shimmer (%)**	M1	5.225	1.486	5.185	0.011*
	M2	4.295	1.509	3.809	
**APQ (%)**	M1	3.490	1.065	3.352	0.055**
	M2	2.979	1.159	2.613	
**PPQ (%)**	M1	1.052	0.786	0.864	0.001**
	M2	0.759	0.766	0.538	
**NHR**	M1	0.141	0.026	0.140	0.052*
	M2	0.130	0.024	0.127	
**CPP vowel**	M1	22.279	3.039	21.432	0.745**
	M2	22.290	3.090	22.232	
**CPPs vowel**	M1	11.229	2.631	10.341	0.897**
	M2	11.288	2.642	11.303	
**CPP speech**	M1	15.406	1.235	15.050	0.603**
	M2	15.365	1.017	15.116	
**CPPs speech**	M1	4.865	1.225	4.580	0.112**
	M2	4.990	1.078	4.803	

* Paired t-test;

** Wilcoxon test

Caption: F0 = fundamental frequency; APQ = amplitude perturbation quotient; PPQ = period perturbation quotient; NHR = noise harmonic ratio; CPP = cepstral peak prominence; CPPs = cepstral peak prominence-smoothed; SD = Standard deviation

Table 2 shows that the analysis of the aerodynamic measures before and immediately after the use of the incentive spirometer indicated higher maximum expiratory volume.

**Table 2:** Aerodynamic measures in the moments before (M1) and immediately after (M2) use of do incentive spirometer

		N	Mean	SD	Median	*p*-value
Maximum expiratory volume(l)	M1	22	2.972	0.867	3.040	0.022*
	M2	22	3.164	0.979	3.275	
Maximum speech time (s)	M1	22	12.923	3.228	12.180	0.890*
	M2	22	12.991	3.337	12.325	
Peak air pressure – cm H_2_O	M1	21	8.590	1.460	8.210	0.580*
	M2	21	8.439	1.802	7.900	
Mean peak air pressure – cm H_2_O	M1	21	7.141	1.249	7.090	0.750*
	M2	21	7.243	1.547	6.790	
Voice airflow – l/s	M1	21	0.118	0.083	0.110	0.632**
	M2	21	0.124	0.093	0.100	
Peak expiratory flow – l/s	M1	21	0.282	0.169	0.240	0.888**
	M2	21	0.347	0.454	0.260	
Aerodynamic resistance – cm H_2_O/ (l/s)	M1	21	65.750	33.257	59.380	0.121**
	M2	21	72.606	45.727	59.000	
Aerodynamic force – W	M1	20	0.097	0.066	0.083	0.401**
	M2	21	0.103	0.084	0.088	
Acoustic impedance – Ω	M1	20	68.114	34.437	60.815	0.113**
	M2	21	74.236	46.445	60.160	
Aerodynamic efficiency – p.p.m.	M1	21	171.45	167.75	90.75	0.271**
	M2	20	245.76	230.11	179.20	

* Paired t-test;

** Wilcoxon test

Caption: SD = Standard deviation

The auditory perception of speech pointed out that half of the participants, that is, 11 women, presented no difference in the voice quality before and after intervention (Table 3).

**Table 3:** Comparison of the auditory perception of speech in the moments before (M1) and immediately after (M2) the use of the incentive spirometer

Auditory perception of speech	Comparison before and after N = 22	
	N	%
Better	8	36.4
Worse	3	13.6
Unchanged	11	50.0

## DISCUSSION

Over the years, respiratory training has been used by physiotherapists in the recovery of patients with several pulmonary pathologies, in addition to preventing pulmonary impairment in the post-surgical period^(10)^. It is recommended that the incentive spirometer is not used isolated but always associated with some conventional technique^(10)^.

Recently, speech therapists have shown interest in using respiratory training devices in voice therapy based on the close relationship between breathing and voice^(5)^. Despite the little scientific evidence available in the literature, respiratory stimulants, either through inhaling or exhaling, have been used for the direct approach of aerodynamic force^(5)^. Research studies performed using the exhalation respiratory training device by Shaker® demonstrated an improvement in voice acoustics and self-perception in dysphonic and non-dysphonic subjects^(17)^, better maximum speech time^(18)^, and fewer laryngeal and vocal symptoms^(19)^.

The market provides several brands and models of respiratory stimulants. The incentive spirometer used in this study, Respiron®, has six different models, ranging from Kids to Athletic 3, with increasing degrees of difficulty and ascending muscle effort requirements^(15)^. Each model has four degrees of adjustment ranging from zero to three^(15)^. This study chose the Classic model due to its level of mean demand and for being the most suitable for the population studied: women, non-singers, and without pulmonary impairment. The Respiron® is considered a training device for inspiratory flow and does not allow accurate verification of the level of pressure exerted during the task performed by the participants. However, for each level of regulation, the manufacturers (NCS S.A., Barueri, SP, Brazil) estimate an appropriate pressure (cm H_2_O) required to perform the task, as follows: approximately 15 cm H_2_O to raise the three spheres at zero regulation (first regulation); 25 cm H_2_O at regulation one (second regulation), 30 cm H_2_O at regulation two (third regulation) and 40 cm H_2_O at regulation three (fourth regulation) (15). Most of the participants in this study (54.6%) used the zero setting (15 cm H_2_O), which suggests lower respiratory conditioning.

The comparison of the acoustic measures before (M1) and after (M2) the use of the device showed significant differences regarding the perturbation measures of on a short term (Table 1): jitter, shimmer, and PPQ, that is, those that express the aperiodicity of the produced sound wave. Variations in the exhaled airflow throughout the speech process might affect the infraglottic pressure and readjust the laryngeal adductor muscles, even interfering with voice intensity and the length of the closing phase of glottal cycles for a harmonic control of glottal resistance (GR)^(20)^. Higher intensities and glottal cycles with longer closing phases tend to manifest through a better conversion of aerodynamic energy into acoustic energy, which reduces the perturbations in the conversion process and optimizes aperiodicity measures, such as those mentioned^(5,20)^. Therefore, considering that the incentive spirometer used herein is an airflow-driven device during the inspiration process, it might have influenced the infraglottic pressure indirectly during the sounded exhalation, which was enough magnitude for the perturbation measures of sound wave generated to change.

In contrast, no significant changes in fundamental frequency were found (Table 1). The factors directly involved in changing the fundamental frequency of a voice are associated with both anatomical issues, like glottal proportion, and physiological issues, such as the contraction mechanisms of the laryngeal muscles and viscoelastic properties of the vocal cords^(20,21)^. Such a finding was expected because the task requested for the use of the incentive spirometer was not associated with speech, thus not directly activating the laryngeal muscles, except for the posterior cricoarytenoid, responsible for the abduction of the vocal cords for the aerodynamic flow to move^(20,22)^.

Likewise, there were no significant variations in the acoustic measures directly linked to the measurement of the harmonic component of the voice: NHR, CPP, and CPPS, both for the speech task and the sustained vowel (Table 1). It is known that throughout glottal cycles, the larger and more uniform the muco-undulatory movement of vocal cords the more harmonic the sound wave generated, positively influencing the values of the mentioned measures^(1,20,23)^. Nonetheless, the stimulation of the mucus wave movement requires the presence of glottal cycles generated in the phonatory process, with the medialization of vocal cords and hence sound by adding the exhaled airflow^(1,20)^. Therefore, such acoustic measures depend on an adequate synergism between the mucus wave movement of vocal cords and subglottic and pulmonary pressures^(1,20)^.

We emphasize that the task requested was to inhale intensely enough to suspend the three spheres of the incentive spirometer used for approximately three seconds. This requires a free and intense airflow transit, and the vocal cords must be in an abduction position without any sound occurring. Therefore, there is no stimulation of muco-undulatory movement. It is likely that inspired airflow training alone, without the presence of sound, was not sufficient to stimulate synergism between the myoelastic and aerodynamic forces of voice production, which could positively impact acoustic measures of NHR, CPP, and CPPS.

Respiratory measures can be classified as volume, time, pressure, flow, and other measures linked to the function^(8)^.

Lung volume, measured in liters, refers to how much air the lung is capable of storing, and is linked not only to the size of the rib cage but also to the capacity for expansion and mobility of the thoracoabdominal wall. It is measured by the task of deep inspiration followed by forced expiration, requiring greater three-dimensional movement of the rib cage^(24)^. It is understood that deeper breaths will favor greater storage of air to be exhaled, resulting in higher lung volume values than shallower breaths due to their relationship with thoracoabdominal movement^(24)^. It is thought that by using the incentive spirometer, the several deep breaths demanded have contributed to making the difference in lung volume significant.

Higher pulmonary volumes demand a lower energy expenditure in speech by favoring the occurrence of the oscillating mechanism of vocal cords^(25)^. In contrast, scenarios involving lower pulmonary volumes decrease the oscillation rates, resulting in higher energy expenditure for the emission to continue, which might also demand greater efforts to increase the fundamental frequency^(25)^.

Maximum speech time is an aerodynamic measure widely used in vocal practice. Recently, it has been introduced in respiratory physiotherapy as a measure of lung function, which represents the maximum time that the subject can sustain the emission, usually the vowel /a/, without the need for another respiratory recharge^(26)^. This measurement is strongly affected by the adduction of vocal cords, since the greater the closure and the longer the duration of the closed phase of the glottal cycles during vocalization, the longer the maximum sustaining time of this emission^(27)^. The adduction of vocal cords is directly linked to the myoelastic force of voice production^(1,20)^. Herein, it is characterized as a distal or secondary target of the intervention using the respiratory stimulant, considering that its primary target is the gain of aerodynamic force. The distal gains promoted by an intervention are believed to require more time to manifest themselves. Thus, for a better understanding of the results of this respiratory stimulant in the treatment of dysphonia, there should be further longitudinal studies, such as clinical trials (Phase 3)^(13)^.

The pressure of a system can be defined as the force acting on a given area. Thus, the greater the compression of particles in a space, such as the infraglottic, the greater the pressure exerted on the walls that delimit such a space, like the vocal cords^(28)^. Therefore, respiratory pressure is high when there is a large volume of air in a given airway space^(1)^. In contrast, airflow is defined as the rate at which air passes through a given point along the respiratory tract. In voice production, it is the variation and interaction between these two measures that allow the self-sustained oscillatory movement of the mucosa covering the vocal cords^(28)^. Measures of respiratory flow and pressure are important and provide the speech-language pathologist with relevant data on the voice production process, specifically regarding aerodynamic force. In addition, they are part of the recommendation protocol for instrumental voice analysis suggested by ASHA (American Speech-Language-Hearing Association)^(6)^.

The incentive spirometer used here is thought to be a device that acts directly on airflow training by requiring that the rate of air flowing inside it is high enough to generate a mechanical force capable of lifting the spheres that compose it. It is worth noting that in this situation the pressure on the walls of the spheres was also verified, being equally fundamental in generating the driving force. After using the incentive spirometer for seven consecutive days, 29 healthy subjects showed a significant increase in inspiratory and expiratory pressure^(11)^.

Thereby, it is expected that the use of the respiratory stimulant promotes positive changes in such measures, thus contrasting with our findings since no significant changes in either pressure or flow were found, considering that the effects of the incentive spirometer were analyzed immediately after (Table 2). Factors such as a shorter intervention time and the number of series repetitions might have influenced our findings. Thus, different combinations of these variables should be investigated to better understand the effects of the studied device.

Some aerodynamic measures provide functional information on the respiratory system, such as resistance, impedance, force, and aerodynamic efficiency^(8)^. They can be classified as functional when manifesting the interaction between pressure and airflow during the respiratory task, associating them with some conversion factor in their algorithms. As mentioned, there was no significant variation in the values of pressure and flow measures before and after the use of the device. In addition, considering that the conversion factors are constant, it was expected that no significant changes in the derived functional measures were found.

Despite the lack of studies addressing the correlation between aerodynamic measures and voice^(12)^, the interface between aerodynamic measures and voice quality is thought to be fundamental not only to the speech therapy clinical practice but also to the follow-up of patients with pulmonary alterations. In this sense, research involving artificial intelligence has aimed to monitor the voice of patients for early detection of exacerbations of diseases such as chronic obstructive pulmonary disease (COPD)^(29)^.

The auditory perception of speech for voice quality is one of the most important tools in the speech therapy clinical practice, both in the evaluation process for decision-making and prognosis establishment and in therapeutic evidence on the vocal techniques selected for the recovery process or vocal conditioning^(30)^. The use of the incentive spirometer improved the voice quality in eight (36.4%) participants, in addition to having not changed the voice quality of half of the participants (50.0%). We believe that since the women in the study had been evaluated as non-dysphonic, the changes in voice quality are more difficult to notice because the phonatory system is already in a homeostasis state^(3)^. Therefore, further studies should include dysphonic subjects (Phase 2)^(13)^.

The literature^(5)^ suggests including vocal respiratory training in the voice treatment to optimize the airflow, in addition to favoring lower overload in the laryngeal muscles during speech and preventing compensatory respiratory patterns of laryngeal disorder from being installed, making the phonatory system more balanced^(5)^. Thereby, the use of other stimulants, such as Shaker® and Threshold®, in both inhalation and exhalation training models also presented the following results: positive effects on the acoustic measure of jitter and speech self-perception^(17)^; reduced laryngeal and vocal symptomatology^(19)^; longer maximum speech time^(18)^ with the use of Shaker®, and positive effects on the aerodynamic measures of pressure and volume with the use of Threshold®^(5)^.

Our results suggest that it is safe to use the incentive spirometer since it showed no negative influence on any of the respiratory or vocal measures assessed, in addition to a positive immediate effect on the acoustic measures (jitter, shimmer, and PPQ) and maximum expiratory volume.

It is worth highlighting that a limitation of this study is the small sample size. In addition, the sample of vocally healthy women did not allow detection of the effects caused by the respiratory stimulant in the process of restoration of homeostasis in the phonatory system since it was already balanced.

Further studies should be conducted using designs that favor higher evidence levels, such as Randomized Clinical Trials, in addition to including subjects with vocal, behavioral, or organic alterations to support evidence-driven practice more expressively.

## CONCLUSION

The immediate effect of using an incentive spirometer is sage and has a positive influence on short-term aperiodicity measures of frequency and intensity acoustic measures, in addition to increasing the maximum expiratory volume in vocally healthy women.
