# Supplementary material for: Immediate effects of the incentive spirometer in women with healthy voice
Source: Codas. 2023 Nov 10;36(1):e20220291. doi: 10.1590/2317-1782/20232022291en (PMC10715584; doi:10.1590/2317-1782/20232022291en)
Supplement: Translation

# Translation: Efeitos imediatos do inspirômetro de incentivo em mulheres vocalmente saudáveis

**DOI:** 10.1590/2317-1782/20232022291pt

**Authors:** Bárbara Pereira Lopes, Gustavo Polacow Korn, Flávio Barbosa Nunes, Ana Cristina Côrtes Gama

**Affiliations:** 1 Programa de Pós-graduação em Ciências Fonoaudiológicas, Universidade Federal de Minas Gerais - UFMG - Belo Horizonte (MG), Brasil.; 2 Departamento de Otorrinolaringologia, Faculdade de Medicina, Universidade Federal de São Paulo - UNIFESP - São Paulo (SP) Brasil.; 3 Departamento de Otorrinolaringologia, Faculdade de Medicina, Universidade Federal de Minas Gerais - UFMG - Belo Horizonte (MG), Brasil.; 4 Departamento de Fonoaudiologia, Faculdade de Medicina, Universidade Federal de Minas Gerais - UFMG - Belo Horizonte (MG) Brasil.

**Keywords:** Voz, Respiração, Exercícios Respiratórios, Fonoaudiologia

## Abstract

**Objetivo:**

Avaliar o efeito imediato do inspirômetro de incentivo nas medidas acústicas, medidas aerodinâmicas e na avaliação perceptivo-auditiva da qualidade vocal de mulheres com vozes saudáveis.

**Método:**

Trata-se de um estudo experimental de comparação intrassujeito que contou com a participação de 22 mulheres sem queixas vocais. Foram obtidas as medidas acústicas, medidas aerodinâmicas e a avaliação perceptivo-auditiva da qualidade vocal antes e imediatamente após o uso do inspirômetro de incentivo pelas participantes. O dispositivo foi utilizado na posição ortostática, e as participantes realizaram três séries de dez repetições com intervalo de um minuto entre as séries.

**Resultados:**

Após uso do inspirômetro de incentivo, observou-se redução significativa nas medidas de *jitter, shimmer* e PPQ (*period perturbation quociente)* e aumento do volume expiratório máximo. As demais medidas acústicas e aerodinâmicas não foram impactadas significativamente. Além disso, houve melhora na avaliação perceptivo-auditiva da qualidade vocal em oito (36,4%) participantes, e 11 (50,0%) não apresentaram mudanças após uso do inspirômetro de incentivo.

**Conclusão:**

O uso do inspirômetro de incentivo é seguro e, em seu efeito imediato, promove redução nas medidas acústicas de aperiodicidade a curto prazo, tanto relacionadas à frequência quanto à intensidade, e aumenta o volume expiratório máximo em mulheres com vozes saudáveis.

## INTRODUÇÃO

A produção da voz pode ser definida como um mecanismo complexo que envolve a fonte sonora, composta pelas pregas vocais propriamente ditas e o trato vocal. O trato vocal funciona como um filtro modulador do som, sendo então o responsável pelos aspectos ressonantais e articulatórios da emissão vocal^(1)^. Além disso, para que a sonorização seja produzida na fonte sonora, faz-se necessária a interação entre duas forças: a aerodinâmica, que se constitui na corrente de ar expirada pelos pulmões e que flui ao longo da traqueia em direção às vias aéreas superiores, e a mioelástica, que se refere ao movimento de medialização das pregas vocais a fim de promover resistência ao ar expirado^(1)^.

A ocorrência dessa resistência à passagem do fluxo aéreo desencadeia um aumento da pressão na região inferior às pregas vocais. Quando elevada, essa pressão é capaz de romper o bloqueio promovido pelas pregas vocais em posição mediana e permitir a passagem do ar por entre elas^(2)^. Logo após esse escape aéreo, a pressão infraglótica diminui novamente, facilitando o retorno da glote ao estado de fechamento e promovendo uma movimentação vibratória da mucosa que recobre as pregas vocais. Esse fenômeno é denominado Efeito Bernoulli, que consiste na produção de deslocamento de matéria devido à diferença de pressão ao longo de um segmento^(1)^. Assim, há a conversão da energia aerodinâmica em energia acústica, resultando em ondas sonoras que são moduladas ao longo de todo o trato vocal^(1)^. Cada vez que esse evento ocorre, há um ciclo glótico, e a ocorrência de vários desses ciclos compõem a produção vocal^(2)^.

Alterações existentes nas forças aerodinâmica e mioelástica, isoladas ou simultâneas, podem causar desequilíbrio no sistema fonatório e comprometer uma emissão vocal saudável em razão da quebra da homeostase^(3)^. Portanto, ambas as forças envolvidas na fonação devem ser consideradas nos momentos de se promover, prevenir e recuperar a saúde da voz.

Cabe ao fonoaudiólogo que atua na clínica vocal oferecer tratamento aos agravos vocais já instalados, ou mesmo aperfeiçoar os ajustes e promover um melhor condicionamento da musculatura envolvida na produção da voz^(4)^. Para tanto, utilizar uma abordagem que contemple não apenas a musculatura laríngea, mas que também considere a respiração envolvida no processo fonatório é de extrema importância para o sucesso terapêutico^(5)^.

Com o passar dos anos, pesquisadores desenvolveram instrumentos capazes de avaliar objetiva e subjetivamente a produção vocal. A extração das medidas acústicas e aerodinâmicas realizadas por meio de *softwares* específicos são exemplos de avaliações objetivas, enquanto a avaliação perceptivo-auditiva e os protocolos de autoavaliação vocal são exemplos de avaliações subjetivas^(6)^. Tais avaliações fomentaram a elaboração de *guidelines* que norteiam os profissionais fonoaudiólogos quanto às suas aplicabilidades, favorecendo a compreensão do funcionamento do sistema fonatório e a identificação de desequilíbrios na interação das forças mioelásticas e aerodinâmicas da produção vocal^(6)^.

O protocolo de avaliação vocal sugerido pela literatura^(6,7)^ compreende a avaliação perceptivo-auditiva da voz, autoavaliação vocal e avaliação instrumental da voz. Esta envolve a avaliação laríngea, a avaliação aerodinâmica da respiração e a análise acústica da voz^(6)^.

Algumas mensurações respiratórias objetivas, como aquelas relacionadas à pressão, volume e fluxo aéreo, podem ser realizadas na avaliação fonoaudiológica, já que a produção vocal se trata de um fenômeno físico envolvendo tais propriedades e seus derivados^(6,8)^. Essas medições são realizadas por *softwares* específicos e extraídas de forma automática por meio de tarefas pré-estabelecidas pelo próprio *software,* semelhante ao que ocorre na extração das medidas acústicas. Elas podem ser úteis não apenas para verificar a integridade do sistema fonatório, mas também podem sugerir patologias pneumológicas, como a asma^(9)^, além de auxiliar no monitoramento da evolução da fonoterapia ao longo dos tratamentos^(5)^.

A inclusão da avaliação respiratória na clínica vocal pode contribuir de forma valiosa na elaboração de um planejamento terapêutico mais assertivo, visto que alguns casos de disfonia evidenciaram uma necessidade de intervenção direta no processo aerodinâmico^(5)^. Essa abordagem direta por meio do treino respiratório pode favorecer um melhor prognóstico, já que esse treino auxilia no reequilíbrio das forças envolvidas no processo de produção vocal^(10)^.

Na prática fonoaudiológica, uma das maneiras de se treinar a musculatura respiratória se dá com o uso de incentivadores respiratórios, dispositivos que envolvem as funções de inspiração e/ou expiração a depender do objetivo terapêutico^(5)^. A literatura^(11)^ relata que o uso do incentivador respiratório, mais precisamente do inspirômetro de incentivo, é capaz de promover aumento nas medidas de pressão inspiratória e expiratória máximas, podendo impactar de forma positiva até mesmo na biomecânica da deglutição.

Incentivadores respiratórios são amplamente utilizados na clínica vocal, porém, o número reduzido de estudos que evidenciaram o impacto de seu uso na clínica vocal^(12)^ assumiu uma função motivacional para o desenvolvimento deste estudo. Compreender a pesquisa científica não apenas como norteadora de uma prática baseada em evidência, mas também como uma aliada à clínica para que sejam confirmados os reais benefícios dessa prática é algo fundamental à ciência, definida então como pesquisa translacional^(13)^.

A literatura^(13)^ classifica as pesquisas experimentais na área da Ciência da Reabilitação em cinco fases: 1) Fase 0, compreende pesquisas observacionais que objetivam definir a prevalência e variáveis associadas a uma determinada condição clínica; 2) Fase 1, são pesquisas experimentais com delineamento de sujeito único sem a condição clínica para avaliar a segurança e efeito da dose de uma determinada intervenção clínica; 3) Fase 2, são pesquisas experimentais com delineamento de sujeito único com a condição clínica para analisar o efeito da intervenção no quadro clínico do sujeito de pesquisa; 4) Fase 3, são estudos experimentais do tipo Ensaios Clínicos Randomizados (ECR) para avaliar a eficácia de uma intervenção; 5) Fase 4, são estudos em população heterogênea sobre a efetividade de uma intervenção e sua aplicabilidade em políticas públicas^(13)^.

Considerando que as pesquisas de Fase 1 são importantes para fomentar o desenvolvimento de estudos futuros com níveis de evidência mais elevados, esta pesquisa apresentou como pergunta norteadora: qual o efeito e a segurança do uso do inspirômetro de incentivo de forma isolada na qualidade da voz e nas medidas aerodinâmicas de indivíduos vocalmente saudáveis?

Portanto, o objetivo deste estudo foi avaliar o efeito imediato do inspirômetro de incentivo nas medidas aerodinâmicas e vocais de mulheres vocalmente saudáveis. Como hipótese de pesquisa, acredita-se que o uso do inspirômetro de incentivo, como uma abordagem respiratória direta, é seguro e passível de ser utilizado na clínica fonoaudiológica, com efeitos positivos na qualidade vocal e nas medidas aerodinâmicas, possivelmente por favorecer uma melhor conversão de energia aerodinâmica em energia acústica, com consequente melhora no desempenho vocal dos sujeitos.

Esta pesquisa justifica-se pelo fato de apresentar conhecimentos científicos sobre a segurança e efeitos imediatos do uso do inspirômetro de incentivo na clínica vocal, subsidiando pesquisas futuras sobre sua eficácia terapêutica por meio de delineamentos com maiores níveis de evidência, como os ensaios clínicos randomizados.

## MÉTODO

Trata-se de um estudo experimental de comparação intrassujeito (Fase 1)^(13)^, aprovado pelo comitê de ética da Universidade Federal de Minas Gerais- UFMG (4.331.770). O delineamento de sujeito único sem a condição clínica (Fase 1) foi definido com base na necessidade de se compreender a segurança e efeito do Respiron® na qualidade vocal e respiração de mulheres vocalmente saudáveis.

Participaram do estudo 22 mulheres, mediante a assinatura do Termo de Consentimento Livre e E sclarecido (TCLE), com idade entre 18 e 43 anos e média de 26 anos (desvio padrão 7,28). A coleta de dados ocorreu no Observatório de Saúde Funcional em Fonoaudiologia da UFMG (OSF/UFMG) em uma sala acusticamente tratada.

Foram incluídas no estudo mulheres sem queixas vocais autorreferidas e com qualidade vocal neutra (G0). A presença ou ausência de queixas vocais foram avaliadas no dia da coleta por meio da análise da autopercepção da qualidade vocal (referir ter/não ter voz boa ou muito boa), que foi positiva, e por meio da análise da presença/ausência de sintomas vocais (fadiga e/ou desconforto), que foi ausente. A qualidade vocal foi analisada pela avaliação perceptivo-auditiva do parâmetro de grau geral da disfonia (G). A avaliação perceptivo-auditiva foi realizada por consenso entre dois fonoaudiólogos com mais de dez anos de experiência em clínica vocal.

Como critérios de exclusão, foram considerados: ser cantora, ser tabagista, ter sentido desconforto na realização dos exercícios e ter obtido pontuação superior a dois pontos na Escala de Dispneia: *Medical Research Council* Modificada, adaptada ao português brasileiro^(14)^.

A Escala de Dispneia é um instrumento de fácil aplicabilidade e compreensão, que avalia de forma gradual em que intensidade a queixa de dispneia impacta na realização das atividades de vida diária^(14)^. Na ausência de queixa de dispneia, as participantes foram orientadas a responder “não se aplica”, e na presença de queixa, deveriam assinalar, dentre um total de cinco itens, qual o impacto desta em suas atividades de vida diária de acordo com as descrições de exemplos oferecidas pela escala^(14)^.

Todas as participantes tiveram suas emissões gravadas em uma sala acusticamente tratada. As emissões foram processadas diretamente em um computador da marca Dell®, modelo Optiplex GX260, com placa de som profissional da marca Direct Sound®. Foi utilizado um microfone unidirecional, condensador, da marca Shure®, posicionado a cinco centímetros da comissura labial, com ângulo de captação direcional de 45°. Os sinais foram gravados a uma taxa de amostragem de 44100 Hz, 16 bits de resolução, mono canal e formato WAV (*Waveform Audio File Format*). Foi solicitado à participante que, em posição ortostática, emitisse a vogal sustentada /a/ de forma habitual e plena, além da contagem de um a 20.

### Avaliações

Cada uma das participantes foi submetida a duas avaliações. A primeira avaliação no momento (M1) foi considerada como o início do período sem intervenção, a medida basal. A segunda avaliação no momento (M2) foi realizada após as participantes utilizarem o inspirômetro de incentivo da marca Respiron® (NCS S.A., Barueri, SP, Brasil) para análise do efeito imediato da técnica (Figura 1).

**Figura 1:** Fluxograma das etapas envolvidas no estudo

#### Avaliação acústica da voz

Para a avaliação acústica, por meio da emissão da vogal /a/, foram consideradas as seguintes medidas extraídas de forma automática pelo programa *Dimensional Voice Program* (MDVP), Kay Pentax®: frequência fundamental, em Hertz; *jitter (%)*, *period perturbation quociente* (PPQ) (*%*), *shimmer (%)* e *amplitude perturbation quotient (*APQ) (*%*), que são medidas de perturbação do sinal sonoro a curto prazo, sendo as duas primeiras referentes às perturbações de frequência e as duas seguintes referentes às perturbações de intensidade; *noise harmonic ratio* (NHR) (dB), que contrasta o componente harmônico e o componente ruído da onda sonora produzida. Também foram extraídas as medidas cepstrais: *Cepstral Peak Prominence* (CPP) (dB) e *Cepstral Peak Prominence-Smoothed* (CPPS) (dB) nas emissões da vogal /a/ sustentada e da amostra de fala obtida na contagem de um a 20 do programa Praat (Paul Boersma e David Weenink, *University of Amsterdam*, Holanda).

#### Avaliação perceptivo-auditiva da qualidade vocal

Para a avaliação perceptivo-auditiva da qualidade vocal, as vozes dos momentos M1 e M2, tanto referentes à vogal sustentada quanto à tarefa de fala por meio da contagem de um a 20, foram dispostas em pares e em ordem aleatória para que os fonoaudiólogos avaliadores apresentassem cegamento em seus julgamentos. Para tanto, três juízes com experiência de mais de cinco anos na clínica vocal foram orientados a classificar os pares de vozes, indicando se houve melhora, piora ou inalteração da qualidade vocal da segunda voz em relação à primeira. Para a realização do procedimento, solicitou-se o uso de fones de ouvido supra-auriculares. Uma vez que as vozes haviam sido classificadas previamente como neutras, ou seja, com desvio no grau geral da qualidade vocal considerado como zero, os juízes não foram demandados quanto à especificação do parâmetro perceptivo-auditivo que mais motivaram suas decisões.

#### Avaliação aerodinâmica da voz

A avaliação das medidas aerodinâmicas foi realizada com uso do *software* CSL da Kay Pentax^TM^, model 6103, Lincoln Park NJ USA, módulo *Phonatory Aerodynamic System* (PAS) por meio de três tarefas: expiração máxima forçada, emissão da vogal /a/ pelo máximo de tempo possível e repetição da sílaba /pa/ por um mínimo de sete vezes consecutivas. Delas foram extraídas as medidas de volume expiratório máximo (litros, l); tempo máximo de fonação (segundos, s); pico de pressão aérea e média do pico de pressão aérea (centímetros de água, cm H_2_O); fluxo aéreo vozeado e pico de fluxo expiratório (litros por segundo, l/s); resistência aerodinâmica [centímetros de água dividido pela fração litros por segundo, cm H_2_O/ (l/s)]; impedância acústica (ohms, Ω); potência aerodinâmica (watts, W) e eficiência aerodinâmica (partes por milhão, p.p.m.)

Para a captação das medidas aerodinâmicas, utilizou-se uma máscara facial de silicone com pequeno cateter de polietileno posicionado sob a língua da participante e acoplada a um transdutor de pressão (Figura 2).

**Figura 2:** Uso da máscara facial de silicone acoplada a um transdutor de pressão para captação das medidas aerodinâmicas

### Utilização do inspirômetro de incentivo da marca Respiron® modelo *Classic*

As participantes foram orientadas a realizar o uso do inspirômetro de incentivo da marca Respiron® modelo *Classic* (NCS S.A., Barueri, SP, Brasil) na posição ortostática (Figura 3). O dispositivo contém três esferas com densidades diferentes e, além disso, o aparelho pode ser ajustado em quatro níveis crescentes de resistência aérea. Nesse caso, o aparelho foi regulado no nível mais elevado com conforto autorrelatado pela participante.

**Figura 3:** Uso do inspirômetro de incentivo da marca Respiron® modelo *Classic*

As orientações oferecidas para o uso do dispositivo consistiram em: expirar o ar ao máximo por via oral; em seguida, inspirar o ar por via oral através do bocal que compõe o dispositivo, tentando elevar as três esferas e mantê-las elevadas por, aproximadamente, três segundos.

Foram realizadas três séries de dez repetições com intervalo de um minuto entre as séries. Após completar o treino, as participantes foram reavaliadas com os mesmos procedimentos utilizados na avaliação inicial. A dose do Respiron® (NCS S.A., Barueri, SP, Brasil), ou seja, o número de repetições, foi definido com base na quantidade sugerida pelo fabricante no manual de uso^(15)^, dose também observada na literatura disponível^(11)^.

### Análise dos dados

A análise estatística dos dados foi realizada por meio do programa estatístico MINITAB versão 17. Primeiramente, foi realizada uma análise descritiva dos dados com medidas de tendência central e dispersão. Posteriormente, foi utilizado o teste de Anderson-Darling para verificar a normalidade da amostra. Para comparação entre os grupos, utilizou-se o teste T pareado ou teste não paramétrico de Wilcoxon. Considerou-se o nível de confiança de 95%. Para análise da concordância intra-avaliadores da avaliação perceptivo-auditiva, foi utilizado o coeficiente AC1 de Gwet no *software* R versão 3.3.1. O grau de concordância foi analisado considerando-se: valores inferiores a zero - concordância ausente; 0 a 0,20 - concordância pequena; de 0,21 a 0,40 - concordância fraca; de 0,41 a 0,60 - concordância moderada; de 0,61 a 0,80 - concordância boa e de 0,81 a 1,00 - concordância quase perfeita^(16)^. Nessa análise, considerou-se o valor da moda das respostas dadas pelos três juízes fonoaudiólogos. Houve discordância entre os três juízes em apenas dois pares de vozes e, nesses casos, um quarto avaliador, fonoaudiólogo especialista em voz com mais de 20 anos de experiência na área, avaliou os dois pares de vozes para a definição da resposta mais concordante.

## RESULTADOS

Para a realização da tarefa orientada, o inspirômetro de incentivo foi regulado conforme o nível mais elevado em que a participante apresentasse conforto por meio do autorrelato. Para tanto, houve uma repetição de teste no nível zero do aparelho, o que também ocorreu nos níveis ascendentes até que se localizasse o nível máximo com presença de conforto autorrelatado. Entretanto, mesmo na presença de autorrelato de conforto, ao se verificar presença de contração exagerada na musculatura cervical, os pesquisadores optavam pelo retorno ao nível anteriormente testado a fim de evitar prejuízos vocais derivados do excesso de tensões musculoesqueléticas. Dessa forma, respeitando-se o nível de conforto na realização da tarefa, 12 participantes (54,6%) utilizaram o dispositivo regulado no nível zero; cinco participantes (22,7%) no nível um, e cinco (22,7%), no nível dois.

Na análise das medidas acústicas nos momentos pré e imediatamente após o uso do inspirômetro de incentivo, observou-se melhora dos parâmetros de aperiodicidade de frequência a curto prazo (*jitter* e PPQ) e de aperiodicidade de intensidade a curto prazo (*shimmer*) (Tabela 1).

**Tabela 1:** Medidas acústicas nos momentos pré (M1) e imediatamente após (M2) o uso do inspirômetro de incentivo (N = 22)

		**Média**	**DP**	**Mediana**	**p-valor**
F0 (Hz)	M1	218,49	27,50	218,95	0,171*
	M2	224,75	27,99	222,83	
Jitter (%)	M1	1,684	0,931	1,461	˂0,001**
	M2	1,147	0,759	1,000	
*Shimmer* (%)	M1	5,225	1,486	5,185	0,011*
	M2	4,295	1,509	3,809	
APQ (%)	M1	3,490	1,065	3,352	0,055**
	M2	2,979	1,159	2,613	
PPQ (%)	M1	1,052	0,786	0,864	0,001**
	M2	0,759	0,766	0,538	
NHR	M1	0,141	0,026	0,140	0,052*
	M2	0,130	0,024	0,127	
CPP vogal	M1	22,279	3,039	21,432	0,745**
	M2	22,290	3,090	22,232	
CPPs vogal	M1	11,229	2,631	10,341	0,897**
	M2	11,288	2,642	11,303	
CPP fala	M1	15,406	1,235	15,050	0,603**
	M2	15,365	1,017	15,116	
CPPs fala	M1	4,865	1,225	4,580	0,112**
	M2	4,990	1,078	4,803	

* Teste T pareado;

** Teste de Wilcoxon

Legenda: F0 = frequência fundamental; APQ = *amplitude perturbation quotient*; PPQ = *period perturbation quotient*; NHR = *noise harmonic ratio*; CPP = *Cepstral Peak Prominence*; CPPs = *Cepstral Peak Prominence-Smoothed*; DP = Desvio padrão

Na Tabela 2, a análise das medidas aerodinâmicas nos momentos pré e imediatamente após o uso do inspirômetro de incentivo indicou aumento do volume expiratório máximo.

**Tabela 2:** Medidas aerodinâmicas nos momentos pré (M1) e imediatamente após (M2) o uso do inspirômetro de incentivo

		N	média	DP	Mediana	P valor
Volume expiratório máximo (l)	M1	22	2,972	0,867	3,040	0,022*
	M2	22	3,164	0,979	3,275	
Tempo máximo de fonação (s)	M1	22	12,923	3,228	12,180	0,890*
	M2	22	12,991	3,337	12,325	
Pico de pressão aérea - cm H_2_O	M1	21	8,590	1,460	8,210	0,580*
	M2	21	8,439	1,802	7,900	
Média do pico de pressão aérea - cm H_2_O	M1	21	7,141	1,249	7,090	0,750*
	M2	21	7,243	1,547	6,790	
Fluxo aéreo vozeado - l/s	M1	21	0.118	0,083	0,110	0,632**
	M2	21	0,124	0,093	0,100	
Pico de fluxo expiratório- l/s	M1	21	0,282	0,169	0,240	0,888**
	M2	21	0,347	0,454	0,260	
Resistência aerodinâmica - cm H_2_O/ (l/s)	M1	21	65,750	33,257	59,380	0,121**
	M2	21	72,606	45,727	59,000	
Potência aerodinâmica - W	M1	20	0,097	0,066	0,083	0,401**
	M2	21	0,103	0,084	0,088	
Impedância acústica - Ω	M1	20	68,114	34,437	60,815	0,113**
	M2	21	74,236	46,445	60,160	
Eficiência aerodinâmica - p.p.m.	M1	21	171,45	167,75	90,75	0,271**
	M2	20	245,76	230,11	179,20	

* Teste T pareado;

** Teste de Wilcoxon

Legenda: DP = Desvio padrão

Na avaliação perceptivo-auditiva da qualidade vocal, verificou-se que metade das participantes, ou seja, 11 mulheres, não apresentaram diferença na qualidade vocal pré e pós intervenção (Tabela 3).

**Tabela 3:** Comparação da análise perceptivo-auditiva da voz nos momentos pré (M1) e imediatamente após (M2) o uso do inspirômetro de incentivo

**Avaliação perceptivo-auditiva**	**Comparação antes e após**		
	**N = 22**		
	**N**	**%**	
Melhorou	8	36,4	
Piorou	3	13,6	
Inalterado	11	50,0	

## DISCUSSÃO

Ao longo dos anos, o treino respiratório tem sido utilizado pelos profissionais da fisioterapia a fim de reabilitar pacientes com diversas patologias pneumológicas e prevenir comprometimentos pulmonares em momentos pós-cirúrgicos^(10)^. Quando utilizado algum inspirômetro de incentivo, a recomendação é que seu uso não seja realizado de forma isolada, mas sempre associado a alguma técnica convencional^(10)^.

Recentemente, os fonoaudiólogos têm demonstrado interesse em utilizar os dispositivos de treino respiratório na clínica vocal devido à estreita relação entre respiração e voz^(5)^. Apesar da pouca evidência científica disponível na literatura, os incentivadores respiratórios, sejam eles de inspiração ou expiração, têm sido utilizados para a abordagem direta da força aerodinâmica^(5)^. Pesquisas realizadas com dispositivo de treino respiratório de expiração da marca Shaker® evidenciou melhora acústica da voz e da autopercepção vocal em sujeitos disfônicos e não disfônicos^(17)^, melhora no tempo máximo de fonação^(18)^ e redução de sintomas laríngeos e vocais^(19)^.

O mercado disponibiliza várias marcas e modelos de incentivadores respiratórios. O inspirômetro de incentivo utilizado neste estudo, o Respiron®, possui seis modelos diferentes, variando do *Kids* ao *Athletic 3*, com crescentes graus de dificuldade e ascendente exigência de esforço muscular^(15)^. Cada modelo apresenta quatro graus de regulagem que variam de zero a três^(15)^. Este estudo optou pelo modelo *Classic* em razão do seu nível de média exigência e por ser o mais indicado para a população estudada: mulheres, não cantoras e sem comprometimento pulmonar. O Respiron® é considerado um dispositivo de treino do fluxo inspiratório e não permite verificar, com exatidão, o nível de pressão exercido durante a tarefa realizada pelas participantes. Entretanto, para cada nível de regulagem, os fabricantes (NCS S.A., Barueri, SP, Brasil) estimaram um valor aproximado da pressão (cm H_2_O) demandada na realização da tarefa, sendo necessários, aproximadamente, 15 cm H_2_O para se elevar as três esferas na regulagem zero (primeira regulagem); 25 cm H_2_O na regulagem um (segunda regulagem), 30 cm H_2_O na regulagem dois (terceira regulagem) e 40 cm H_2_O na regulagem três (quarta regulagem)^(15)^. A maioria das participantes desta pesquisa (54,6%) utilizou a regulagem zero (15 cm H_2_O), o que sugere um menor condicionamento respiratório.

Comparando-se as medidas acústicas nos momentos pré (M1) e pós (M2), foram observadas diferenças significativas em relação às medidas de perturbação da onda sonora a curto prazo (Tabela 1): *jitter, shimmer* e PPQ, ou seja, aquelas que expressam quão aperiódica é a onda sonora produzida. Variações no fluxo de ar expirado durante o processo de fonação podem alterar a pressão infraglótica e reajustar a musculatura adutora da laringe, interferindo até mesmo na intensidade vocal e na duração da fase fechada dos ciclos glóticos a fim de se obter um controle harmônico da resistência glótica^(20)^. Intensidades mais elevadas e ciclos glóticos com maior duração de fase fechada tendem a se manifestar com uma melhor conversão de energia aerodinâmica em energia acústica, o que reduz as perturbações presentes no processo de conversão e otimiza as medidas de aperiodicidade como as supracitadas^(5,20)^. Dessa forma, considerando que o inspirômetro de incentivo utilizado nesta pesquisa se trata de um dispositivo movido a fluxo de ar durante o processo de inspiração, acredita-se que ele tenha influenciado de maneira indireta a pressão infraglótica durante a expiração fonada, tendo sido essa interferência de magnitude suficiente para que as medidas de perturbação da onda sonora gerada tenham se modificado.

Por outro lado, não foram observadas mudanças significativas na frequência fundamental (Tabela 1). Os fatores envolvidos de forma direta na mudança da frequência fundamental de uma voz estão associados tanto a questões anatômicas, como a proporção glótica, quanto a questões fisiológicas, como os mecanismos de contração da musculatura da laringe e propriedades viscoelásticas das pregas vocais^(20,21)^. Esse achado já era esperado pelo fato de a tarefa solicitada com uso do inspirômetro de incentivo não ter sido associada à fonação, não tendo promovido uma ativação direta da musculatura laríngea, exceto do cricoaritenóideo posterior, responsável pela abdução das pregas vocais a fim de permitir o transitar do fluxo aerodinâmico^(20,22)^.

De forma semelhante, não foram observadas variações significativas nas medidas acústicas diretamente relacionadas à mensuração do componente harmônico da voz: NHR, CPP e CPPS, tanto na tarefa de fala quanto de vogal sustentada (Tabela 1). Já se sabe que, durante a ocorrência dos ciclos glóticos, quanto mais amplo e mais uniforme for o movimento muco-ondulatório das pregas vocais, mais harmônicos serão produzidos pela onda sonora gerada, impactando de forma positiva nos valores das medidas acima citadas^(1,20,23)^. Entretanto, para a estimular o movimento muco-ondulatório, é necessário que haja a presença de ciclos glóticos gerados no processo fonatório onde ocorre a medialização das pregas vocais e consequente sonorização ao se adicionar o fluxo aéreo expirado^(1,20)^. Portanto, tais medidas acústicas são dependentes de um adequado sinergismo entre o movimento muco-ondulatório das pregas vocais e das pressões subglótica e pulmonar^(1,20)^. Ressaltamos que a tarefa solicitada foi a de inspirar de forma intensa o suficiente para suspender, por aproximadamente três segundos, as três esferas do inspirômetro de incentivo utilizado. Isso requer um livre e intenso transitar do fluxo aéreo, devendo as pregas vocais estarem em posição de abdução sem ocorrência de sonorização e não havendo, por conseguinte, estimulação do movimento muco-ondulatório. Provavelmente, o treino do fluxo aéreo inspirado de forma isolada, sem a presença de sonorização, não foi suficiente para estimular o sinergismo entre as forças mioelásticas e aerodinâmicas da produção vocal, o que poderia impactar positivamente nas medidas acústicas de NHR, CPP e CPPS.

As medidas respiratórias podem ser classificadas em medidas de volume, tempo, pressão, fluxo e outras medidas relacionadas à função^(8)^.

O volume pulmonar, medido em litros, refere-se a quanto ar o pulmão é capaz de armazenar, além de se relacionar não apenas ao tamanho da caixa torácica, mas também à capacidade de expansão e mobilidade da parede toracoabdominal. Isso porque, para medi-lo, utiliza-se a tarefa de inspiração profunda seguida de expiração forçada, demandando maior movimentação tridimensional da caixa torácica^(24)^. Entende-se que inspirações mais profundas irão favorecer um maior armazenamento de ar a ser expirado, resultando em valores mais elevados de volume pulmonar quando comparados a inspirações mais superficiais devido a sua relação com a movimentação toracoabdominal^(24)^. Acredita-se que, ao se utilizar o inspirômetro de incentivo, as várias inspirações profundas demandadas tenham contribuído para tornar significativa a diferença do volume pulmonar.

Maiores volumes pulmonares demandam um menor gasto energético durante a fonação, pois favorecem a ocorrência do mecanismo oscilante das pregas vocais^(25)^. Em contrapartida, situações de menor volume pulmonar promovem um decaimento nas taxas de oscilações, resultando em maior gasto energético para que a emissão continue e podendo, inclusive, demandar grandes esforços a ponto de elevar a frequência fundamental^(25)^.

O tempo máximo de fonação é uma medida aerodinâmica amplamente utilizada na clínica vocal. Recentemente, ela foi introduzida na fisioterapia respiratória como medida de função pulmonar, que representa o máximo de tempo que o sujeito é capaz de sustentar a emissão, normalmente a vogal /a/, sem que tenha que fazer uma nova recarga respiratória^(26)^. Essa mensuração é fortemente impactada pela adução das pregas vocais, pois quanto maior o fechamento e maior o tempo de duração da fase fechada dos ciclos glóticos durante a vocalização, maior será o tempo máximo de sustentação dessa emissão^(27)^. A adução de pregas vocais se relaciona de forma direta com a força mioelástica da produção vocal^(1,20)^ e, nesta pesquisa, caracteriza-se como alvo distal ou secundário da intervenção com uso do incentivador respiratório, tendo em vista que seu alvo primário é o ganho da força aerodinâmica. Acredita-se que os ganhos distais promovidos por uma intervenção demandem mais tempo para se manifestarem e, nesse sentido, estudos longitudinais, do tipo ensaios clínicos (Fase 3)^(13)^ são necessários para uma melhor compreensão dos resultados desse incentivador respiratório no tratamento das disfonias.

A pressão de um sistema pode ser definida como a força que atua em uma determinada área. Assim, quanto maior a compressão das partículas num espaço, como a infraglote, maior será a pressão exercida sobre as paredes que delimitam tal espaço, como as pregas vocais^(28)^. Para tanto, a pressão respiratória é alta quando há um grande volume de ar em um determinado espaço das vias aéreas^(1)^. Em contrapartida, o fluxo de ar é definido como a taxa de passagem do ar por um determinado ponto ao longo do trato respiratório. Na produção vocal, é a variação e a interação entre essas duas medidas que permitem o movimento oscilatório autossustentado da mucosa que recobre as pregas vocais^(28)^. Medidas de fluxo e pressão respiratórias são importantes e fornecem ao fonoaudiólogo dados relevantes sobre processo de produção vocal, especificamente referente à força aerodinâmica, e fazem parte do protocolo de recomendação de análise instrumental da voz sugerido pela ASHA (*American Speech-Language-Hearing Association*)^(6)^.

Acredita-se que o inspirômetro de incentivo aqui utilizado seja um dispositivo que atua de forma direta no treinamento do fluxo aéreo por demandar que a taxa de ar que transita em seu interior seja alta o suficiente a ponto de gerar uma força mecânica capaz de elevar as esferas que o compõem . Ressalta-se que nessa situação também verificou-se a atuação da pressão sobre as paredes das esferas, tendo sido igualmente fundamental na geração da força motriz. Um aumento significativo nas medidas de pressão inspiratória e expiratória foi observado em 29 sujeitos saudáveis após uso do inspirômetro de incentivo por sete dias consecutivos^(11)^. Espera-se, portanto, que o uso do incentivador respiratório seja capaz de promover mudanças positivas nessas medidas não corroborando com os achados deste presente estudo no qual não houve mudanças expressivas relacionadas à pressão e fluxo, considerando-se que a análise sobre os efeitos do inspirômetro de incentivo foi imediatamente após o uso (Tabela 2). Fatores como tempo reduzido de intervenção e número de repetições e séries podem ter influenciado os achados. Dessa forma, diferentes combinações dessas variáveis devem ser estudadas para melhor compreensão dos efeitos desse dispositivo.

Ainda referente às medidas aerodinâmicas, existem aquelas que trazem informações funcionais do sistema respiratório: resistência, impedância, potência e eficiência aerodinâmicas^(8)^. Elas podem ser classificadas como funcionais por expressarem como ocorre a interação da pressão e do fluxo de ar durante a tarefa respiratória associando essas duas medidas a algum fator de conversão em seus algoritmos. Conforme já dito anteriormente, não houve variação significativa entre os valores referentes às medidas de pressão e fluxo nos momentos pré e pós uso do dispositivo e, tendo em vista que os fatores de conversão são constantes, já se era esperado, por conseguinte, que não houvesse mudança significativa nas medidas funcionais derivadas.

Apesar da escassez de estudos que explorem a correlação entre medidas aerodinâmicas e voz^(12)^, acredita-se que a interface entre as medidas aerodinâmicas e a qualidade vocal sejam de grande valia não apenas na clínica fonoaudiológica, mas também no acompanhamento de pacientes com alterações pulmonares. Nesse sentido, pesquisas envolvendo a inteligência artificial têm sido desenvolvidas objetivando o monitoramento da voz dos pacientes para a detecção precoce de agravamentos de doenças como a doença pulmonar obstrutiva crônica (DPOC)^(29)^.

A avaliação perceptivo-auditiva da qualidade vocal representa uma das ferramentas mais importantes na clínica fonoaudiológica, tanto no processo avaliativo para tomada de decisão quanto na definição de prognóstico e provas terapêuticas das técnicas vocais selecionadas no processo de reabilitação ou condicionamento vocal^(30)^. O uso do inspirômetro de incentivo melhorou a qualidade vocal de oito (36,4%) participantes, além de não ter alterado a qualidade vocal de metade das participantes (50,0%). Acredita-se que, por se tratar de mulheres previamente avaliadas como não disfônicas, as mudanças na qualidade vocal sejam mais difíceis de serem percebidas, pois o sistema fonatório já se encontra em um estado de homeostase^(3)^. Para tanto, estudos com sujeitos disfônicos (Fase 2)^(13)^ se fazem necessários.

A literatura^(5)^ sugere a inclusão do treino respiratório no tratamento vocal a fim de otimizar o fluxo de ar, além de auxiliar na redução da sobrecarga da musculatura laríngea durante a fonação e na prevenção da instalação de padrões respiratórios compensatórios a uma disfunção laríngea, tornando o sistema fonatório mais equilibrado^(5)^. Nesse sentido, a utilização de outros incentivadores, como o Shaker® e o Threshold® nos modelos de treino da inspiração ou da expiração, também resultou em: efeitos positivos na medida acústica de *jitter* e na autopercepção vocal^(17)^; redução da sintomatologia laríngea e vocal^(19)^; aumento no tempo máximo de fonação^(18)^ no uso do Shaker® e efeitos positivos nas medidas aerodinâmicas de pressão e volume no uso do Threshold®^(5)^.

Os resultados desta pesquisa sugerem que a utilização do inspirômetro de incentivo é segura, já que não impactou de forma negativa em nenhuma das medidas respiratórias e vocais avaliadas, além de ter apresentado um efeito imediato positivo nas medidas acústicas (*jitter, shimmer* e PPQ) e no volume expiratório máximo.

Ressalta-se como limitação do estudo o reduzido tamanho amostral e, por se tratar de uma amostra de mulheres vocalmente saudáveis, não foi possível verificar os efeitos que o uso do incentivador respiratório desencadeia no processo de reestabelecimento da homeostase do sistema fonatório, tendo em vista que este já se encontrava equilibrado.

São necessários estudos futuros com delineamentos que favoreçam maiores níveis de evidência, como os ensaios clínicos randomizados, e que incluam sujeitos com alterações vocais, de base comportamental ou orgânica, a fim de fomentar de forma mais expressiva a prática baseada em evidência.

## CONCLUSÃO

O uso do inspirômetro de incentivo, em seu efeito imediato, é seguro e impacta positivamente as medidas acústicas de aperiodicidade a curto prazo de frequência e intensidade, além de aumentar o volume expiratório máximo em mulheres com vozes saudáveis.
